# Gout inheritance in an extended Chinese family analyzed by whole-exome sequencing: A case-report

**DOI:** 10.1097/MD.0000000000020057

**Published:** 2020-06-19

**Authors:** Peiqing Yang, Xuenan Pi, Tony N. Marion, Jing Wang, Gang Wang, Yan Xie, Dan Xie, Yi Liu

**Affiliations:** a Department of Rheumatology; b Precision Medicine Key Laboratory of Sichuan Province & Precision Medicine Center, West China Hospital, Sichuan University, Chengdu, Sichuan, P.R. China; c Department of Microbiology, Immunology and Biochemistry, University of Tennessee Health Science Center, Memphis, Tennessee.

**Keywords:** Gout, low-density lipoprotein receptor-related protein 1, oncoprotein induced transcript 3, whole-exome sequencing

## Abstract

**Introduction:**

Gout is a worldwide chronic disease generally caused by high serum levels of uric acid. Using whole exome sequencing, we aimed to explore genetic alterations in hereditary gout.

**Patients’ concerns:**

There were 9 direct descendants diagnosed with gout in total in this family. The patients concerned about the high incidence and inheritance of gout.

**Diagnosis:**

The youngest propositus was diagnosed as gout in our hospital. Diagnoses of other patients in this family were made on the foundation of history and clinical tests.

**Interventions:**

Six direct descendants and 3 healthy spouses in 1 family were recruited in our study. Whole-exome sequencing was conducted in all participants.

**Outcomes:**

Whole-exome sequencing and genetic analysis revealed 2 putative rare inherited deleterious variants, which were detected only in direct descendants. Twelve gout and uric acid (UC)-related nucleotide sequence variants previously reported by GWAS were detected among all subjects.

**Conclusions:**

In the case of this family, the GWAS identified gout and UC-related nucleotide sequence variants may increase the risk of developing gout, but penetrance was not complete. The rare sequence variants in low-density lipoprotein receptor-related protein 1 (LRP1) and oncoprotein induced transcript 3 (OIT3) may have contributed to inheritance of gout within the 5 generations of family members in this study.

## Abstract 2

Supplemental Digital Content is available in the text

## 1 Introduction

Gout is a chronic metabolic disease characterized by elevated level of uric acid in serum, together with acute inflammatory arthritis caused by sedimentation of monosodium urate crystals in joints and synovial tissue. The incidence of gout is generally 2 to 6 times higher in men than in women and increases with age, reaching a plateau after the age of 70. Noticeably, more affluent countries have a higher rate of gout compared with less affluent ones.^[1]^ In China the prevalence has been estimated to be 1.14% in adults.^[2,3]^ Both non-hereditary and hereditary factors play a role in the development of gout. Non-genetic risk factors include age, sex, diet, and drugs. Genetic research has identified a number of loci associated with gout and serum levels of uric acid (UC) in the population.^[4–7]^ Sequence variants of MUC1, SLC17A1, GCKR, ABCG2, and SLC2A9 have all been associated with gout and/or elevated serum UC, but heredity for gout remains incompletely understood.

In this study, we analyzed the exome data of a family with a pattern of inherited gout. The family members were screened for both genetic risk loci and rare inherited sequence variants.

## 2 Case report

### 2.1 Subjects description

**Figure 1:**
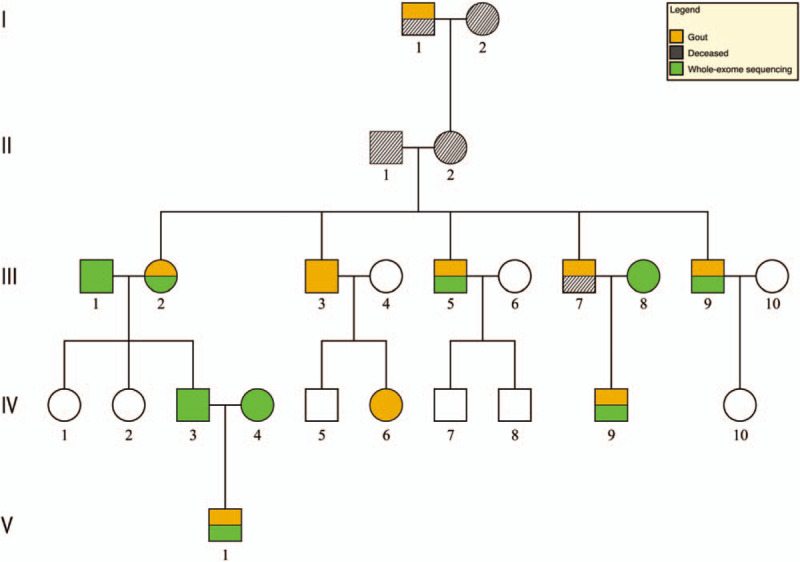
Family pedigree and diagnosis of gout.

In total, there were 9 family members diagnosed with gout (Fig. 1). All the patients met the 2015 diagnostic criteria for gout classification.^[8]^ Detailed information about clinical features were collected from hospital records or by questionnaire and reviewed by experienced physicians. All subjects gave their written consent to participate in the study, and the study was approved by the ethics committee.

The first identified member with gout was I-1. The youngest identified, V-1, was identified in our hospital. V-1 manifested gout on left toes 4 years ago with blood uric acid level 417 μmol/L, and suffered iterative gout flare 1 to 2 times a year in the following 4 years. One year ago, this patient had pain and tenderness in his left knee with blood uric acid 395 μmol/L. There has been no clear distinction in diet among the family members. More detailed information of all gout patients in this family is in Table 1.

**Table 1:**
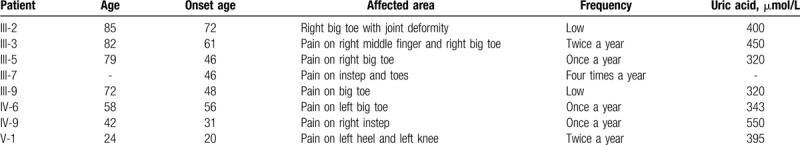
Clinical information of gout patients.

### 2.2 DNA extraction and whole exome sequencing

EDTA anti-coagulated venous blood (5 mL) was collected from each included subject. 250 ng of genomic DNA from each individual was sheared by Biorupter (Diagenode, Belgium) to acquire 150–200 bp fragments. Illumina Sequencing adaptors were added to repaired DNA fragment ends (Fast Library Prep Kit, iGeneTech, Beijing, China). After constructing the sequencing library, the whole exomes were captured with AIExome Enrichment Kit V1 (iGeneTech, Beijing, China) and sequenced on the NovaSeq Illumina platform (Illumina, San Diego, CA) with 150 base paired-end reads.

### 2.3 Detection of variant, GWAS loci, and susceptible inherited rare variants

The Illumina 150 bp paired end reads was trimmed by fastp (v0.19.4). Trimmed reads were mapped to human reference genome (GRCh37/hg19) by BWA MEM (v0.7.17). Nucleotide sequence variants were identified by following GATK (v4.0.10) best practice. Twenty nine WES samples from the 1000 Genome Project were genotyped together with the family data, so as to fulfill the requirement of at least 30 samples in the VariantRecalibrator step. Variant annotation was conducted by Annovar (Apr 16, 2018).

Information for the GWAS loci related to gout/uric acid was obtained from the NHGRI GWAS catalog (Jan 29, 2019) (Supplementary Table S1, genomic coordinate in GRCh38). Only the loci with CI and Beta information and from East Asian population data were considered relevant.

Exome-wide SNPs and INDELs were filtered in the following analytical sequence under both recessive and dominant inherited model (in-house python script): variants within intronic region or synonymous function annotation; variants with 1000 genome population frequency >0.5% or ExAC non-TCGA East Asian population frequency >0.5%; SIFT score >0.5 or Polyphen2 HDIV score <0.5 or FATHMM score >0.

### 2.4 Whole-exome sequencing analysis

Considering the hereditary pattern for gout in the family, we hypothesized that inheritance of genetic susceptibility contributed to gout in the III to V generation descendents. Six direct descendants and 3 related spouses in total agreed to participate in the whole-exome sequencing test: III-1, III-2, III-5, III-8, III-9, IV-3, IV-4, IV-9, and V-1.

**Figure 2:**
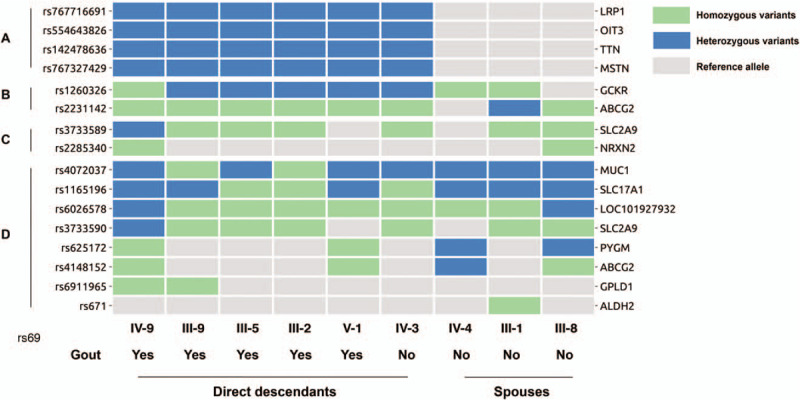
Detected GWAS loci and susceptible inherited rare variants in the family members. (A) Susceptible inherited rare variants; (B) GWAS loci related to both uric acid and gout in East Asian population; (C) GWAS loci related to gout only in East Asian population; (D) GWAS loci related to uric acid only in East Asian population; Rs ID of the variants are displayed on the left side; names of genes that each variant located in are displayed on the right side.

Firstly, we analyzed the genomic sequences for potentially inherited polymorphisms or mutations that might exist only in direct descendants but not spouses. Based upon the dominant inherited phenotype model,^[9]^ 4 missense variants that were not previously reported to have association with gout or UC were identified (Fig. 2A and Table 2). Among the 4 variants, rs767716691 (T1376N) within *lrp1*, 12q13.3, and rs554643826 (T439S) within *oit3*, 10q22 have potential associations with gout or UC.^[10,11]^ According to the gnomAD database (r2.0.2), both variants were only found with low frequency in an East Asian population as heterozygous alleles (Table 2). CADD phred score indicated that the *lrp1* and *oit3* variants were in the top 1% of deleterious variants in the human genome (Table 2): *lrp1* was in the top 0.14% most intolerant genes, and *oit3* was in the top 28.67% most intolerant genes based on RVIS values (Table 2). It is possible these 2 mutations of low-density lipoprotein receptor-related protein 1 (LRP1) and oncoprotein induced transcript 3 (OIT3) could contribute to the development of gout in the direct descendants.

**Table 2:**
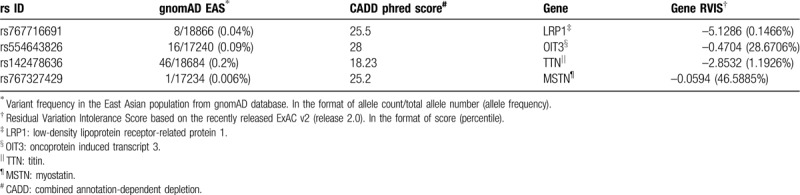
Inheritance of the A variants in Fig. 2 among the general East Asian population.

We also analyzed GWAS identified gout and UC-related single nucleotide polymorphisms (SNP) in the family members to check for inheritance of previously identified gout and UC-related polymorphisms. To account for genetic polymorphisms that distinguish individuals in the East Asian population in general, we focused on genetic loci that have been previously reported in the East Asian population. Among 68 East Asian polymorphisms associated with gout, 13 were covered by the exome enrichment kit used in this study (see Supplementary Table S1). Twelve out of the 13 polymorphisms were detected in the subjects (Fig. 2B–D). Ten variants that were detected both in the direct descendants and the spouses, include rs1260326, rs2231142, rs3733589, rs2285340, rs4072037, rs1165196, rs6026578, rs3733590, rs625172, and rs4148152. rs6911965 was detected in 2 of the direct descendants, and rs671 was detected in 1 of the spouses. Homozygous versus heterozygous inheritance of East Asian gout and/or UC associated SNP among family descendants and spouses are indicated in Fig. 2.

## 3 Discussion

Gout and hyperuricemia are becoming an increasing heath problem in both developed and developing countries.^[1]^ It has been identified that hyperuricemia is associated with metabolic syndrome (MetS) such as hyperglycemia, hypertension, obesity, and dyslipidemia. Hyperglycemia is a notable risk factor for hyperuricemia, and insulin resistance may play a crucial role in this process.^[12]^ In addition, increased serum uric acid is significantly correlated with hypertriglyceridemia and low high-density lipoprotein cholesterol (HDL-C).^[13]^ A conceivable mechanism is that triglyceride synthesis strengthens the de novo synthesis of ribose-5-phosphate to phosphoribosyl pyrophosphate (PPRP) via the NADP-NADPH pathway, which increases the uric acid production.^[14]^

We have analyzed the inheritance of gout in 5 generations within 1 family. Whole-exome sequencing results suggest that inherited genetic variation may be associated with gout in this family. Two rare, highly detrimental variants, LRP1(rs767716691) and OIT3(rs554643826) were detected in 6 direct descendants, although 1 of the 6 descendants (IV-3) has not been diagnosed with gout. The absence of gout in the descendent who inherited the genetic variants simply confirms the reality that gout is a complex disease that may be influenced by many poorly understood factors. The rare occurrence of the 2 sequence variants associated with gout within a single family and the inheritance pattern over 5 generations within that family provide both support and potential insight about the relevance of LRP1(rs767716691) and OIT3(rs554643826) to gout. Certainly these results support further continued research about the role of LRP1 and OIT3 in gout susceptibility.

The LRP1 is a type I transmembrane protein belonging to the low-density lipoprotein receptor (LDL-R) family. LRP1 is capable of binding with >40 unrelated ligands, such as the α2-macroglobulin-protease complex (α2M) and triglyceride-rich lipoprotein-derived apolipoprotein E (apoE).^[15]^ LRP1 is also a crucial regulator of intracellular cholesterol accumulation in macrophages and smooth muscle cells (SMCs)^[16,17]^ and serves to export redundant cholesterol out of the cell.^[18]^ In addition, the endocytosis and intracellular transport of LRP1 plays a predominant role in regulating the functions and activities of other receptors and plasma membrane proteins that molecularly interact with LRP1.^[19]^ This multifunctional receptor is widely expressed on many tissues and cells including liver, brain, muscle, monocyte/macrophages, and fibroblasts.^[20]^ LRP1 plays a vital role in glycol-metabolism and lipoprotein metabolism, and has been verified in relation to the features of MetS.^[21,22]^ This receptor could regulate the insulin signaling and glucose metabolism through interacting with insulin receptor β (IRβ) and modulating the level of glucose transporters, GLUT3 and GLUT4. LRP1 deficiency reduced both GLUT3 and GLUT4 levels in neuronal cells and impaired brain insulin signaling.^[23]^ As an endocytic receptor, LRP1 also has the capacity to regulate lipid metabolism. This receptor enhances the uptake of TG-rich lipoproteins in an insulin-dependent way through interaction with apoE.^[24]^ Inactivation of LRP1 in liver resulted in a significant HDL-cholesterol (HDL-C) decrease in serum,^[25]^ and previous studies indicate an indirect regulation of LRP1 in HDL-C production.^[24]^ Given that increased level of serum UC is substantially correlated with hypertriglyceridemia and low HDL-C,^[13]^ LRP1 gene may potentially regulate the concentration of serum UC and indirectly play a role in the development of gout. The mechanism is considered to be functionally associated with insulin-resistance and glucose/lipoprotein metabolism.^[14]^

Other studies have provided evidence for the role that LRP1 plays in gout. LRP1 was reported to be a potential gene genetically correlated with INHBC/INHBE locus.^[26]^ Inhibins and activins are members of the transforming growth factor β superfamily. Noticeably, INHBB/INHBC/INHBE and ACVR1B/ACVRL1 inhibin-activin encoding genes have been associated with serum urate loci.^[27]^ Another intriguing study elucidated the structural and functional relationship between LRP1 and LRP2, which was associated with serum UC in a GWAS among East Asians.^[28]^ All these findings suggest that LRP1 may affect the progression of gout by regulating the expression of gout and UC-related genes.

The oncoprotein induced transcript 3 (OIT3), also known as liver-specific zona pellucida domain-containing protein (LZP), generally functions as a secreted protein.^[29,30]^ OIT3 was identified to be involved in renal uric acid excretion.^[11]^ Intriguingly, Yan et al^[11]^ discovered that OIT3 null mice have reduced serum uric acid and enhanced excretion of this metabolite. So far there is no direct evidence elucidating the effects of mutations in OIT3 in humans, but a mutation in an 8 cysteines conserved domain in LZP was associated with familial juvenile hyperuricemia nephropathy.^[9]^

As to the inheritance in the family of gout and UC associated SNP previously identified by GWAS, no definitive inheritance pattern was found. The 12 gout and UC-related SNP previously identified by GWAS were inherited among the family members but without obvious prevalence in the direct descendants with gout. On the one hand, there is a natural limitation of the covered region of the whole-exome enrichment kit, only 13 out of 68 loci are captured (Supplementary Table S1). In fact, in the 68 selected loci, 58 are within intronic, UTR or intergenic regions (Supplementary Table S1). Exome sequencing can only capture exonic and part of intronic regions near exons. On the other hand, GWAS comparison on such a small sample size would be futile. The insight to be gained from this study of inheritance of gout susceptibility is in the identification of 2 additional loci hereto fore not discovered to be relevant in gout by GWAS that may warrant future attention.

## Author contributions

**Conceptualization:** Yi Liu.

**Data curation:** Peiqing Yang, Jing Wang.

**Formal analysis:** Peiqing Yang, Xuenan Pi.

**Investigation:** Peiqing Yang, Xuenan Pi.

**Methodology:** Xuenan Pi.

**Project administration:** Gang Wang.

**Software:** Yan Xie, Dan Xie.

**Supervision:** Yi Liu.

**Visualization:** Peiqing Yang.

**Writing – original draft:** Peiqing Yang.

**Writing – review & editing:** Tony Marion.

## Supplementary Material

Supplemental Digital Content
